# Supplementary material for: Micronutrient Status of Critically Ill Patients with COVID-19 Pneumonia
Source: Nutrients. 2024 Jan 29;16(3):385. doi: 10.3390/nu16030385 (PMC10856879; doi:10.3390/nu16030385)
Supplement: Supplementary file 1 [file nutrients-16-00385-s001.zip › Supplementary File S1.pdf]

Supplementary File S1

Enteral nutrition AMC

Peptamen® Intense: Peptamen Intense | Target Nutrition App from Nestlé

Enteral nutrition VUmc

Peptamen® Intense: Peptamen Intense | Target Nutrition App from Nestlé

Peptamen® HN: Peptamen HN Peptide Feed | Nestlé Health Science (nestlehealthscience.co.uk)

Fresubin® 2 KCAL HP: Fresubin® 2 KCAL HP (FIBRE) - Fresenius Kabi Netherlands (fresenius-kabi.com)

Nutrison® Advanced Protison: Nutrison Protein Advance (nutricia.co.uk)

Nutrison® Protein Intense: Nutrison Protein Intense (nutricia.co.uk)

Nutrison® Protein Plus: Nutrison Protein Plus (nutricia.co.uk)

Daily administration of additional micronutrients in VUmc

Table 1: Cernevit

Vitamin A	3500 IU
Biotin	69 µg
Folic acid	414 µg
Vitamin B12	6 µg
Thiamine	3.51 mg
Riboflavin	4.14 mg
Vitamin B6	4.53 mg
Panthotenic acid	17.25 mg
Vitamin C	125 mg
Vitamin D	220 IU (D3)
Vitamin E	10.2 mg

Table 2: Supliven

Chlorium chloride hexahydrate	53.3 µg
Potassium jodide	166 µg
Cupper chloride dihydrate	1.0 mg
Manganese chloride tetra hydrate	0.198 mg
Sodium fluoride	2.1 mg
Sodium molybdate dihydrate	48.5 µg
Sodium selenite anhydrate	173 µg
Ferric chloride hexahydrate	5.4 mg
Zinc chloride	10.5 mg

Table 3: Supradyn complex forte

Vitamin A	800 µg
Vitamin B1	3,3 mg
Vitamin B2	4,2 mg
Vitamin B3	48 mg
Vitamin B5	18 mg
Vitamin B6	2 mg
Vitamin B8	50 µg
Folic acid	200 µg
Vitamin B12	3 µg
Vitamin C	180 mg
Vitamin D	5 µg
Vitamin E	12 mg
Vitamin K	25 µg
Calcium	120 mg
Iron	14 mg
Jodine	150 µg
Copper	1 mg
Magnesium	80 mg
Manganese	2 mg
Molybdenum	50 µg
Selenium	50 µg
Zinc	10 mg
