# Supplementary material for: Micronutrient Status of Critically Ill Patients with COVID-19 Pneumonia
Source: Nutrients. 2024 Jan 29;16(3):385. doi: 10.3390/nu16030385 (PMC10856879; doi:10.3390/nu16030385)
Supplement: Supplementary file 1 [file nutrients-16-00385-s001.zip › Supplementary File S3.pdf]

Supplementary File S3 - Prevalence of deficiency

Vitamin A (<1.2 µmol/L)				
	Baseline	Week 1	Week 2	Week 3
**AMC**	100%	30%	30%	20%
**VUmc**	100%	70%	10%	30%
**Controls**	0% (0/5)			

Vitamin B6 (<35 nmol/L)				
	Baseline	Week 1	Week 2	Week 3
**AMC**	80%	90%	100%	80%
**VUmc**	100%	80%	80%	90%
**Controls (n=3)**	100% (3/3)			

Vitamin D (<50 nmol/L)				
	Baseline	Week 1	Week 2	Week 3
**AMC**	70%	50%	60%	60%
**VUmc**	70%	70%	70%	70%
**Controls**	60% (3/5)			

Vitamin E (<15 µmol/L)				
	Baseline	Week 1	Week 2	Week 3
**AMC**	10%	0%	10%	0%
**VUmc**	10%	10%	10%	0%
**Controls**	0% (0/5)			

Iron (<11 µmol/L)				
	Baseline	Week 1	Week 2	Week 3
**AMC**	90%	100%	90%	100%
**VUmc**	100%	100%	90%	90%
**Controls**	40% (2/5)			

Transferrin (<1.6 g/L)				
	Baseline	Week 1	Week 2	Week 3
**AMC**	70%	70%	50%	60%
**VUmc**	90%	80%	50%	30%
**Controls**	0% (0/5)			

Zinc (<10 µmol/L)				
	Baseline	Week 1	Week 2	Week 3
**AMC**	80%	60%	30%	10%
**VUmc**	70%	70%	50%	30%
**Controls**	0% (0/5)			

Selenium (<0.70 µmol/L)				
	Baseline	Week 1	Week 2	Week 3
**AMC**	50%	30%	10%	0%
**VUmc**	60%	0%	0%	0%
**Controls**	0% (0/5)			

Copper (<10 µmol/L)				
	Baseline	Week 1	Week 2	Week 3
**AMC**	0%	0%	0%	0%
**VUmc**	0%	0%	0%	0%
**Controls**	0% (0/5)			
